# Patient- and treatment-related risk factors associated with neck muscle spasm in nasopharyngeal carcinoma patients after intensity-modulated radiotherapy

**DOI:** 10.1186/s12885-017-3780-9

**Published:** 2017-11-23

**Authors:** Lu-Lu Zhang, Guan-Qun Zhou, Zhen-Yu Qi, Xiao-Jun He, Jia-Xiang Li, Ling-Long Tang, Yan-Ping Mao, Ai-Hua Lin, Jun Ma, Ying Sun

**Affiliations:** 1 Department of Radiation Oncology, Sun Yat-sen University Cancer Center, State Key Laboratory of Oncology in South China, Collaborative Innovation Center for Cancer Medicine, 651 Dongfeng Road East, Guangzhou, 510060 People’s Republic of China; 2 Department of Clinical Medicine, School of Public Health, Sun Yat-sen University, Guangzhou, People’s Republic of China; 3 Department of Oncology, First People’s Hospital of Zhaoqing City, Guangdong, People’s Republic of China; 4 Department of Medical Statistics and Epidemiology, School of Public Health, Sun Yat-sen University, Guangzhou, People’s Republic of China

**Keywords:** Nasopharyngeal carcinoma, Neck muscle spasm, Intensity-modulated radiotherapy, Dose tolerance

## Abstract

**Background:**

To evaluate the incidence of neck muscle spasm in nasopharyngeal carcinoma (NPC) patients that received intensity-modulated radiotherapy (IMRT), and to analyse the patient- and treatment-related risk factors associated with neck muscle spasm.

**Methods:**

A sample of 152 IMRT-treated, biopsy-proven, nondisseminated NPC patients were retrospectively analysed. All had documented IMRT treatment plans and had returned for follow-up review at 4 years post-radiotherapy. Spasm of the sternocleidomastoid (SCM) muscle was graded from 0 to 3 (absent to severe) and this grade served as the clinical endpoint. Risk factors were identified using logistic regression analysis.

**Results:**

Within 4 years of radiotherapy, neck muscle spasm developed in 23.68% of the patients; Grades 0, 1, 2 and 3 were respectively assigned to 83.55, 7.57, 6.58 and 2.30% of assessed SCMs. Multivariate analysis indicated that gender, N stage, V60 (percentage of SCM volume that received >60 Gy) were independent prognostic variables, and that the optimal threshold for using V60 to predict neck muscle spasm was 61.92% (sensitivity = 0.900, specificity = 0.953).

**Conclusions:**

Gender, N stage and V60 were independent predictive factors for post-radiotherapy neck muscle spasm, and a V60 of ≤61.92% in the SCM was relatively safe.

## Background

Nasopharyngeal carcinoma (NPC) represents the most common malignant tumour of the nasopharyngeal epithelium. While relatively rare in western countries, it is more frequently diagnosed in Southeast Asia. The highest incidence is found in Southern China, where the incidence in males can reach 20–50 per thousand. [1]. NPC is one of the most radiosensitive cancers, and radiation therapy (RT) is usually the definitive treatment [2]. In recent years, intensity-modulated radiotherapy (IMRT) has become accepted as a more advanced radiation technique for treatment of NPC [3–5]. With the 5-year overall survival rate for NPC patients treated with IMRT increasing to 79.6% [6], focus has shifted to improving the quality of life of these survivors, who can experience late adverse events such as cervical subcutaneous fibrosis, hearing loss and skin dystrophy [7].

Having the neck muscles present within or adjacent to the high-dose radiation fields is unavoidable for NPC patients. High-dose-radiation induced neck muscle spasm, which has received little attention until recently, is a sudden and involuntary ‘Charlie-horse-like’ contraction of the neck muscles with or without pain. It lasts for seconds to minutes and is concentrated in the sternocleidomastoid (SCM) muscles of head and neck cancer (HNC) patients [8]. It may be triggered by head turning, lifting and yawning, and it can be alleviated by neck stretching or massage. In some HNC patients, the spasm-induced pain is sufficient to require additional interventions such as physical therapy, medication or injection of botulinum-A toxin [8–10]. However, these interventions can only relieve the neck spasms temporarily; therefore, investigating risk factors and developing preventative measures seems a better focus for research.

Previous research has demonstrated a strong dose-response relationship between neck muscle spasm and the radiation dose received by the SCM of HNC patients [9]. However, the independent prognostic variables for post-radiotherapy neck muscle spasm remain unclear; moreover, of the few published studies on the topic, none examined patients with NPC [8–10]. Hence, we carried out this retrospective study to investigate the incidence of post-radiotherapy neck muscle spasm in NPC patients, and to analyse potential clinical and treatment-related risk factors.

## Methods

### Patient selection

This was a retrospective longitudinal cohort study performed at our cancer centre. Between July and September 2011, 267 newly diagnosed, nondisseminated, biopsy-proven NPC patients were treated using IMRT with or without chemotherapy. Patients returned to the hospital for follow-up review at least every 3 months for the first 2 years, and then every 6 months until death. During each follow-up, a detailed history was taken and a thorough physical examination was performed, along with chest radiography and abdominal ultrasonography. Magnetic resonance imaging (MRI) of the neck and nasopharynx was performed every 6 to 12 months.

Of the 267 NPC patients, 37 were excluded owing to the loss of 4-year follow-up results, and 78 were excluded because their IMRT treatment-plan documents were unavailable. In the 152 remaining subjects, the occurrence and severity of neck muscle spasm was ascertained via a phone-based following-up at 4 years post-radiotherapy. This retrospective study was approved by the institutional ethics committee and the need for informed consent was waived.

### Treatment methods

Before treatment, all patients underwent a baseline evaluation, including a thorough history and physical examination, haematology and biochemistry profiles, MRI of the nasopharynx and neck, chest radiography, abdominal ultrasonography, and bone scan emission computed tomography. All patients were staged according to the 7th edition of the AJCC staging system [11].

All patients underwent definitive IMRT with or without chemotherapy. Details concerning the implementation of IMRT at our cancer centre, which complies with reports 50 and 62 of the International Commission on Radiation Units and Measurements, have been reported previously [12–15]. The total radiation doses (delivered in 28–33 fractions) were 66–72 Gy for the primary tumour, 64–70 Gy for the cervical lymph nodes, 60–63 Gy for the high-risk region, and 54–56 Gy for the low-risk and neck nodal regions.

During the study, institutional guidelines recommended only IMRT for stage I and concurrent chemoradiotherapy with or without neoadjuvant/adjuvant chemotherapy for stages II to IVB. Concurrent chemotherapy consisted of cisplatin every one or 3 weeks, and neoadjuvant or adjuvant chemotherapy consisted of three cycles of cisplatin with 5-fluorouracil, or cisplatin with taxanes every 3 weeks. Patients exhibiting persistent disease or relapse underwent salvage treatment procedures such as surgery, chemotherapy and afterloading.

### Data collection

#### Patient- and treatment-related factors

The medical records of the sample group were retrospectively reviewed to collect data concerning potential patient- and disease-related risk factors (gender, age, T stage, N stage, smoking status, drinking status), as well as treatment-related risk factors (dosimetric parameters for the SCM, use of chemotherapy and/or neck surgery). The dosimetric parameters were obtained from dose volume histograms (DVHs) of the SCM. We re-delineated bilateral SCMs according to our previously proposed methods [16] to generate the bilateral neck DVHs for each patient using the CERR DICOM-RT toolbox (version 3.0 beta 3; School of Medicine, Washington University, St. Louis, USA). The following dosimetric parameters were collected: mean dose (Dmean), maximum dose (Dmax), minimum dose (Dmin), percentage of the SCM volume that received more than X Gy (VX), the dose received by X% of the SCM volume (DX); values of X were 20, 25, 30, 35, 40, 45, 50, 55, 60, 65, 70, 75 and 80.

#### Grading of neck muscle spasm to yield study endpoints

Owing to the lack of a universally recognized classification system, we proposed a 4-point scale to score SCM muscle spasm according to the most serious degree of neck muscle spasm in the 4 years post-treatment, as follows: grade 1 for mild SCM spasm occurring infrequently, without pain and/or impaired neck mobility; grade 2 for moderate SCM spasm occurring frequently with contractile pain, but without impaired neck mobility; and grade 3 for severe SCM spasm occurring daily with pain and occasionally also with impaired neck mobility. This grade served as the clinical endpoint.

### Statistical analysis

All statistical analyses were performed using SPSS 13.0 (Chicago, IL, USA) and a two-tailed *P* value of <0.05 was considered statistically significant. For analysis of differences between SCMs without neck muscle spasm and those with it, a χ^2^ test was used for categorical variables and a Wilcoxon rank-sum test was used for continuous variables. Binary logistic regression was used for univariate analyses. Receiver operating characteristic (ROC) curves were generated to estimate the cut-off points for all significant dosimetric parameters in the univariate logistic regression analysis and to create a dose-volume histogram (DVH) for neck muscle spasm. All factors that had a *P* value of <0.05 after univariate logistic regression analysis were included in a multivariate logistic regression analysis to determine the independent factors associated with neck muscle spasm. Receiver operating characteristic (ROC) curve analysis was adopted for selecting optimal cut-off points for independent dosimetric factors that were predictive of neck muscle spasm.

## Results

### Pre-treatment (baseline) characteristics of patients and incidence of neck muscle spasms

Of the 152 NPC patients included in the final study, 114 were men and 38 were woman. Their ages ranged from 14 to 71 years, with the median being 41. The proportion with stage-I, −II, −III and -IV disease were 3/152 (1.97%), 16/152 (10.53%), 67/152 (44.08%) and 66/152 (43.42%), respectively.

Almost all the patients (151/152, 99.34%) were diagnosed with undifferentiated squamous-cell carcinoma (type II) according to the World Health Organization (WHO) classification, and 1 (0.66%) patient was diagnosed with squamous-cell carcinoma (type I). Radiotherapy (RT) alone was used to treat 14 patients (9.21%), while the remaining 137 (90.13%) were treated using chemo-radiotherapy. One patient (0.66%) underwent bilateral neck dissection and 9 (5.92%) underwent unilateral neck dissection after completion of RT.

By 4 years post-IMRT, 36 patients (23.68%) had developed SCM muscle spasms, and among these, there were 22 cases of unilateral spasm and 14 cases of bilateral spasm. Owing to the fact that both right and left SCM muscles were evaluated, a total of 304 (2 × 152) SCMs were included in the study. Most (254; 83.55%) exhibited no spasms, while 23 (7.57%) showed mild spasms, 20 (6.58%) showed moderate spasms and 7 (2.30%) exhibited severe spasms. Of the 36 patients in the current study who developed SCM muscle spasms, no patient underwent medication, and only two patients underwent physiotherapy. Most patients relieved symptoms temporarily by neck stretching or massage.

### Comparison of baseline characteristics of SCMs with spasms to those of SCMs without spasms

A more detailed list of the comparisons is given in Table 1, but the following parameters were found to be significantly different between SCMs with and without spasms: gender, N stage, Dmean, Dmin, Dmax, V20–75 and D20–80. Difference in age, T stage, smoking status, drinking status, induction chemotherapy, concurrent chemotherapy, neck dissection and V80 (*P* = 0.537) were not found to be significant.

**Table 1:** Baseline (pre-treatment) characteristics of SCMs without neck muscle spasm and those with neck muscle spasm

Variables	SCMs without neck muscles spasm	SCMs with neck muscles spasm	*P* value
Group number	254	50	
Sex			0.003
Male	199 (78.35%)	29 (58.00%)	
Female	55 (21.65%)	21 (42.00%)	
Age (years)			0.471
≤ 41	131 (51.57%)	23 (46.00%)	
> 41	123 (48.43%)	27 (54.00%)	
T stage			0.356
T1–2	61 (24.02%)	9 (18.00%)	
T3–4	193 (75.98%)	41 (82.00%)	
N stage			0.002
N0–1	127 (50.00%)	13 (26.00%)	
N2–3	127 (50.00%)	37 (74.00%)	
Smoking status			0.799
Yes	86 (33.86%)	16 (32.00%)	
No	168 (66.14%)	34 (68.00%)	
Drinking status			0.851
Yes	38 (14.96%)	8 (16.00%)	
No	216 (85.04%)	42 (84.00%)	
Induction chemotherapy			0.839
Yes	123 (48.43%)	25 (50.00%)	
No	131 (50.57%)	25 (50.00%)	
Concurrent chemotherapy			0.851
Yes	216 (85.04%)	42 (84.00%)	
No	38 (14.96%)	8 16.00%)	
Neck dissection			0.324
Yes	8 (3.15%)	3 (6.00%)	
No	246 (96.85%)	47 (94.00%)	
D mean	50.54 Gy (38.04 Gy – 58.91 Gy)	62.05 Gy (61.22 Gy – 62.35 Gy)	< 0.001
D min	2.68 Gy (1.04 Gy – 29.44 Gy)	35.88 Gy (30.35 Gy – 39.58 Gy)	< 0.001
D max	68.82 Gy (66.24 Gy – 71.46 Gy)	70.80 Gy (68.58 Gy −72.51 Gy)	0.048
V20^a^	84.94% (61.87% – 100%)	100% (100% – 100%)	< 0.001
V25	81.70% (59.84% – 100%)	100% (100% – 100%)	< 0.001
V30	79.52% (57.88% – 99.99%)	100% (100% – 100%)	< 0.001
V35	77.57% (56.23% – 99.76%)	100% (99.88% – 100%)	< 0.001
V40	75.57% (54.40% – 98.99%)	99.89% (99.22% – 100.00%)	< 0.001
V45	74.00% (52.43% – 96.44%)	99.07% (97.67% – 99.70%)	< 0.001
V50	70.87% (47.81% – 90.72%)	96.32% (93.59% – 98.32%)	< 0.001
V55	64.00% (41.94% – 78.86%)	87.74% (84.56% – 91.79%)	< 0.001
V60	39.02% (23.11% – 52.10%)	68.33% 64.01% – 72.25%)	< 0.001
V65	8.16% (0.97% – 20.59%)	35.01% (23.10% – 41.22%)	< 0.001
V70	0.00% (0.00% – 0.76%)	1.66% (0.00% – 13.78%)	< 0.001
V75	0.00% (0.00% – 0.00%)	0.00% (0.00% – 0.00%)	0.010
V80	0.00% (0.00% – 0.00%)	0.00% (0.00% – 0.00%)	0.537
D20^b^	63.12 Gy (60.59 Gy – 65.28 Gy)	66.74 Gy (64.93 Gy – 68.01 Gy)	< 0.001
D25	62.39 Gy (59.74 Gy – 64.31 Gy)	66.04 Gy (64.30 Gy – 67.35 Gy)	< 0.001
D30	61.69 Gy (58.95 Gy – 63.39 Gy)	65.27 Gy (63.61 Gy – 66.61 Gy)	< 0.001
D35	60.76 Gy (57.74 Gy – 62.53 Gy)	64.76 Gy (63.05 Gy – 65.92 Gy)	< 0.001
D40	59.79 Gy (55.88 Gy – 61.70 Gy)	63.97 Gy (62.68 Gy – 64.85 Gy)	< 0.001
D45	59.06 Gy (53.84 Gy – 61.01 Gy)	63.23 Gy (62.31 Gy – 64.05 Gy)	< 0.001
D50	58.17 Gy (49.85 Gy – 60.38 Gy)	62.48 Gy (61.59 Gy – 63.14Gy)	< 0.001
D55	57.52 Gy (38.66 Gy – 59.55 Gy)	61.75 Gy (61.01 Gy – 62.44 Gy)	< 0.001
D60	56.26 Gy (27.73 Gy – 58.83 Gy)	61.10 Gy (60.35 Gy – 61.67 Gy)	< 0.001
D65	54.85 Gy (16.57 Gy – 58.14 Gy)	60.22 Gy (59.61 Gy – 60.86 Gy)	< 0.001
D70	51.52 Gy (9.49 Gy – 57.19 Gy)	59.80 Gy (58.47 Gy – 57.81 Gy)	< 0.001
D75	43.68 Gy (5.57 Gy – 56.15 Gy)	58.42 Gy (57.51 Gy – 59.37 Gy)	< 0.001
D80	35.34 Gy (3.21 Gy – 54.78 Gy)	57.21 Gy (56.01 Gy – 58.29 Gy)	< 0.001

*Abbreviations*: *SCM* sternocleidomastoid muscle, *Dmean* Mean dose to the sternocleidomastoid muscle, *Dmax* Maximum dose to the sternocleidomastoid muscle; V20^a^ is the percentage of the sternocleidomastoid muscle volume that received more than 20 Gy; D20^b^ is the dose to 20% of the sternocleidomastoid muscle volume; the other dosimetric parameters are reported in a similar manner

### Univariate analysis and dose-volume histogram

The univariate logistic regression analysis is described in Table 2 and it showed that gender, N stage, Dmean, Dmin, Dmax, V20–65 and D20–80 were significantly associated with post-radiotherapy SCM spasm. In contrast, there was no significant association with age, T stage, smoking status, drinking status, induction chemotherapy, concurrent chemotherapy, neck dissection, V75 and V80.

**Table 2:** Univariate and multivariate analysis of patient- and treatment-related risk factors for neck muscle spasm

	*P* value	OR (95% CI)
Univariate analysis		
Sex		
Male		Ref
Female	0.003	2.620 (1.387, 4.949)
Age (years)		
≤ 41		Ref
> 41	0.472	1.250 (0.681, 2.297)
T stage		
T1–2		Ref
T3–4	0.358	1.440 (0.662, 3.131)
N stage		
N0–1		Ref
N2–3	0.002	2.846 (1.445, 5.607)
Smoking status		
Yes	0.799	0.919 (0.481, 1.758)
No		Ref
Drinking status		
Yes	0.851	1.083 (0.472, 2.485)
No		Ref
Induction chemotherapy		
Yes	0.839	1.065 (0.581, 1.953)
No		Ref
Concurrent chemotherapy		
Yes	0.851	0.924 (0.402, 2.120)
No		Ref
Neck dissection		
Yes	0.332	1.963 (0.502, 7.671)
No		Ref
D mean	< 0.001	1.002 (1.001, 1.003)
D min	< 0.001	1.001 (1.001, 1.001)
D max	0.007	1.001 (1.000, 1.002)
V20^a^	< 0.001	1.085 (1.045, 1.127)
V25	< 0.001	1.085 (1.046, 1.125)
V30	< 0.001	1.083 (1.046, 1.121)
V35	< 0.001	1.081 (1.045, 1.118)
V40	< 0.001	1.082 (1.046, 1.120)
V45	< 0.001	1.085 (1.048, 1.122)
V50	< 0.001	1.118 (1.068, 1.171)
V55	< 0.001	1.148 (1.096, 1.202)
V60	< 0.001	1.192 (1.133, 1.255)
V65	< 0.001	1.106 (1.076, 1.136)
V70	< 0.001	1.126 (1.072, 1.183)
V75	0.653	1.173 (0.585, 2.349)
V80	0.999	0.000 (0.000, 0.000)
D20^b^	< 0.001	1.004 (1.003, 1.005)
D25	< 0.001	1.005 (1.003, 1.006)
D30	< 0.001	1.005 (1.003, 1.006)
D35	< 0.001	1.005 (1.004, 1.007)
D40	< 0.001	1.005 (1.004, 1.007)
D45	< 0.001	1.005 (1.004, 1.007)
D50	< 0.001	1.005 (1.003, 1.007)
D55	< 0.001	1.003 (1.002, 1.005)
D60	0.001	1.002 (1.001, 1.003)
D65	0.001	1.001 (1.000, 1.002)
D70	< 0.001	1.001 (1.000, 1.001)
D75	< 0.001	1.001 (1.000, 1.001)
D80	< 0.001	1.001 (1.000, 1.001)
Multivariate analysis		
Sex		
Male		Ref
Female	0.024	3.044 (1.157, 8.012)
N stage		
N0–1		Ref
N2–3	0.035	2.823 (1.078, 7.398)
V60	< 0.001	1.185 (1.126, 1.246)

*Abbreviations*: *Dmean* mean dose to the sternocleidomastoid muscle, *Dmax* maximum dose to the sternocleidomastoid muscle, *V20* ^a^ percentage of the sternocleidomastoid muscle volume that received >20 Gy, *D20* ^b^ dose to 20% of the sternocleidomastoid muscle volume; other dosimetric parameters are reported in a similar manner

The significant dosimetric parameters from the regression analysis were included in the ROC curve analysis to identify the dose tolerance cut-off points for SCM spasm. The cut-off points were selected using the Youden index at the level of *P* < 0.05, and were as follows (Table 3): V20 (99.99%), V25 (99.99%), V30 (99.94%), V35 (98.94%), V40 (97.58%), V45 (94.72%), V50 (90.02%), V55 (65.78%), V60 (61.92%), V65 (28.94%) and V70 (0.57%).

**Table 3:** Radiation dose tolerances for the SCM with respect to neck muscle spasm, as determined using ROC curve analysis

	Area under ROC curve	Standard error	*P*	Lower limit	Upper limit	Cut-off point	Sensitivity	Specificity
V20^a^	0.795	0.030	<0.001	0.737	0.853	99.99%	0.920	0.673
V25	0.810	0.029	<0.001	0.752	0.867	99.99%	0.900	0.713
V30	0.815	0.029	<0.001	0.758	0.872	99.94%	0.900	0.709
V35	0.834	0.029	<0.001	0.777	0.892	98.94%	0.920	0.665
V40	0.834	0.029	<0.001	0.777	0.891	97.58%	0.920	0.685
V45	0.826	0.029	<0.001	0.769	0.883	94.72%	0.900	0.689
V50	0.860	0.025	<0.001	0.810	0.910	90.02%	0.900	0.732
V55	0.895	0.023	<0.001	0.849	0.941	65.78%	0.940	0.524
V60	0.934	0.024	<0.001	0.887	0.981	61.92%	0.900	0.953
V65	0.848	0.303	<0.001	0.784	0.912	28.94%	0.680	0.902
V70	0.675	0.046	<0.001	0.584	0.766	0.57%	0.600	0.744

*Abbreviations*: *SCM* sternocleidomastoid muscle, *ROC* receiver operating characteristic, *V20* ^a^ percentage of the sternocleidomastoid muscle volume that received >20 Gy; other dosimetric parameters are reported in a similar manner

**Fig. 1:**
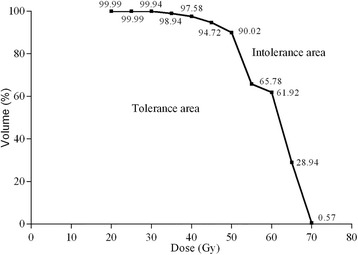
Dose tolerance curves for post-radiotherapy neck muscle spasm in the sternocleidomastoid muscle (SCM). Dose-volume histograms was created using the cut-off points in Table 3. The area under the DVH curve represented tolerable doses for the SCM with respect to post-radiotherapy neck muscle spasm

A DVH was established using the above cut-off points (Fig. 1). The area under the DVH curve represented tolerable doses for the SCM with respect to neck muscle spasm, and the area above the curve represented intolerable doses. As the dose and percentage volume of the SCM increased, the tolerable area gradually reduced, indicating that the probability of neck muscle spasm increased gradually with radiation dose.

### Multivariate analysis

**Fig. 2:**
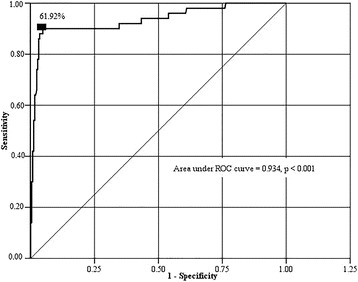
Receiver operating characteristic (ROC) curve for the V60 (percentage of the sternocleidomastoid muscle volume that received more than 60 Gy). A ROC curve was generated to determine the dose tolerance for moderate/severe neck muscle spasm. A V60 of 61.92% had a sensitivity of 0.900 and a specificity of 0.953 and was considered the tolerance dose of the sternocleidomastoid (SCM) muscle with respect to post-radiotherapy spasms. The area under the ROC curve for a V60 of 61.92% was 0.934

After multivariate logistic regression analysis, differences in gender (*P* = 0.024, *β* = 1.113, *SE* = 0.494, *odds ratio [OR]* = 3.044, *95% CI* = 1.157 to 8.012), N stage (*P* = 0.035, *β* = 1.038, *SE* = 0.491, *OR* = 2.823, 95% *CI* = 1.078 to 7.398) and V60 (*P* < 0.001, *β* = 0.169, *SE* = 0.026, *OR* = 1.185, *95% CI =* 1.126 to 1.246) were found to be significant (Table 2). Female gender and an advanced N stage were patient-related risk factors for neck muscle spasm. The ROC curve for V60 is shown in Fig. 2, and the area under the curve was 0.934. The optimal threshold for V60 to predict neck muscle spasm was 61.92% (sensitivity = 0.900 and specificity = 0.953). Among the SCMs without neck muscle spasm, 4.7% received a radiation dose where V60 was >61.92%, while for those with spasm, V60 was >61.92% in 90.0% of cases (*P* < 0.001).

## Discussion

This is the first and largest retrospective study to date to identify the incidence and risk factors for neck muscle spasm in NPC patients treated with IMRT. Analysis of the results identified gender, N stage and V60 as independent risk factors and these findings could be used to aid IMRT planning in NPC patients.

### NPC patients suffered a high incidence of post-radiotherapy neck muscle spasm

Post-radiotherapy neck muscle spasm among HNC patients began to receive attention about two decades ago, however, to date, only three papers have been published regarding this adverse effect in HNC patients. Van Daele et al. first reported the condition in 2002, finding that after RT in the neck area, 9 HNC patients suffered neck muscle spasm, concentrated in the SCM [9]. Then in 2011, Gelblum et al. reported that 14 HNC patients developed severe neck spasm after undergoing IMRT ± chemotherapy [10]. Finally, in 2013, Hunter et al. observed that 9.7% (34/352) of HNC patients complained of radiation-induced bilateral or unilateral neck spasm during follow-up (median, 51 months; range, 30–90 months); with the spasms being especially pronounced in the SCM [8]. The mechanism of postradiation muscle spasm is not clear, but it is likely related to high-dose-radiation-induced and progressive fibrosis-induced ischemia.

In the present study, the occurrence rate of neck muscle spasm among patients with NPC 48 months after RT was 23.68% (36/152); this is more than double the incidence of neck muscle spasm among patients with other types of HNC, as reported by Hunter et al. The discrepancy may be explained as follows: on account of the rich lymphatic network in the nasopharynx, the incidence of cervical-lymph-node metastasis is higher for NPC than for other HNCs [17]. Therefore, irradiation of the neck nodes, along with the entire region of lymphatic drainage, is the standard treatment method [2]. However, neck dissection is the standard procedure for HNC patients with clinically positive neck lymph node metastases [18]. Above all, the radiation dose to the SCM region is higher in NPC patients versus those with other HNCs, and this leads to a higher incidence of post-radiotherapy neck muscle spasm.

### Advanced N stage and female gender were patient-related independent risk factors

We found that being at the advanced N stage was a negative risk factor for neck muscle spasm. This may be due to the fact that advanced N-stage NPC merits an increased dose of radiation to the positive cervical lymph nodes and the region of lymphatic drainage. Therefore, the volume of the SCM and peripheral nerve receiving high-dose radiation is necessarily higher, and this increases the probability of muscle and nerve injury [19].

Studies regarding the relationship between gender and RT-induced late complications in NPC patients remain controversial [20, 21]. Lee et al. found that male gender was a negative risk factor for temporal lobe necrosis, cranial nerve neuropathy, radiation myelitis, osteoradionecrosis and dysphagia in NPC patients [20]. In contrast, Yeh et al. found that female gender was a negative independent predictor of hearing deficits, tinnitus and otorrhea in NPC patients [21]. Our results indicate that female gender is a negative independent risk factor for neck muscle spasm. Although the mechanism of these gender-related differences remains unclear, we speculate that differences in gene expression and hormone secretion between males and females may play an important role.

These findings should prompt us to pay more attention to female patients and advanced N-stage patients during follow-up on account of the higher probability of neck muscle spasm.

### Chemotherapy and neck dissection had no effect on neck muscle spasm

Several studies have shown that combining chemotherapy with RT does not seem to sensitize soft tissue to radiation injury [16, 19, 22]. Consistent with these studies, our results suggest that chemotherapy does not increase the incidence of neck muscle spasm when compared with RT alone.

The findings of earlier studies concerning the association between neck dissection and the development of post-radiotherapy neck muscle spasm have been inconsistent. Hunter et al. found that neck dissection did not increase the risk of post-radiotherapy neck muscle spasm in patients with oropharyngeal cancer [8]. On the other hand, Gelblum et al. reported that neck surgery may increase the incidence of neck muscle spasm for HNC patients following IMRT; however, the study only included a small number of patients, so this conclusion needs to be verified [10]. In our study, we did not observe an effect of neck dissection on muscle spasm. This may be explained by the fact that neck dissection can cause serious damage to SCM muscle innervation, thus hindering the associated neural activity, including the abnormal spontaneous variety.

### V60 was an independent risk factor

Until to now, only Hunter et al. had investigated the association between dose and neck muscle spasm. By comparing (*t*-test) dosimetric parameters between SCMs with and without neck muscle spasm, the authors found that the differences between spasm groups were significant for all such parameters (univariate analysis). Owing to the authors’ belief that Dmean was the most convenient dosimetric parameter to use, they put forward its use in formulating the cut-off points for predicting the occurrence of neck muscle spasm. However, in the current study, Dmean was only significant in the univariate analysis, not in the multivariate analysis. Our study indicated V60 to be the independent dosimetric risk factor. Moreover, our results showed that keeping the SCM’s V60 below 61.92% makes post-radiotherapy neck muscle spasm relatively unlikely.

Currently, the Radiation Therapy Oncology Group (RTOG) protocol recommends 70 Gy to the cervical lymph nodes and 54 Gy to the lymphatic drainage regions [23]. SCMs that were located near to drainage regions, may have suffered a high dose of radiation. However, IMRT provides the ability to deliver excellent target-volume coverage while protecting adjacent normal tissues. Therefore, it may be possible for radiation oncologists to design IMRT plans that keep V60 below 61.92% for the SCM. Of course, the true clinical utility of applying a V60 of 61.92% as the cut-off value for predicting post-radiotherapy neck muscle spasm requires more evidence.

### Limitations

It is worth noting two limitations of the current study. Firstly, the its retrospective nature was unavoidable, but it means that a prospective study will be necessary to validate the findings. Secondly, a longer follow-up period may yield additional conclusions: 4 years may not be long enough. However, in previous studies, the median latency for occurrence of neck muscle spasm ranged from 23 to 37 months, implying that 4 years (48 months) is a reasonable choice. Thirdly, due to the lack of a universally recognized classification system, we proposed a four-point scale to score SCM muscle spasm, which was not previously validated. Evaluation bias may exist due to using such an unvalidated clinician-graded measure as the primary endpoint. Prospective design of studies of patient-reported neck spasm may be required in future research to increase the reliability of the evaluation.

## Conclusions

NPC patients exhibited a high frequency of neck muscle spasm at 4 years post-radiotherapy. The patient-related factors, gender and N stage, and the treatment-related factor, V60, were independent predictors of neck muscle spasm. Moreover, a V60 of 61.92% may represent the tolerance dose for this late post-radiotherapy complication. These findings may help improve risk assessment for neck muscle spasm, and aid the optimization of IMRT treatment plans in NPC patients.

## Abbreviations

Dmax: Maximum dose
Dmean: Mean dose
Dmin: Minimum dose
DVH: Dose-volume histogram
DVHs: Dose volume histograms
DX: the Dose received by X% of the sternocleidomastoid volume
HNC: Head and neck cancer
IMRT: Intensity-modulated radiotherapy
MRI: Magnetic resonance imaging
NPC: Nasopharyngeal carcinoma
OR: Odds ratio
ROC: Receiver operating characteristic
RT: Radiation therapy
RTOG: Radiation Therapy Oncology Group
SCM: Sternocleidomastoid
VX: Percentage of the sternocleidomastoid volume that received more than X Gy
WHO: World Health Organization
